# Complete Genome Sequences of Seven Strains of *Pseudomonas* spp. Isolated from Boreal Forest Soil in Interior Alaska

**DOI:** 10.1128/MRA.00511-20

**Published:** 2020-06-18

**Authors:** Tracie Haan, Taylor J. Seitz, Alexis Francisco, Kaytlin Glinter, Alexander Gloger, Anna Kardash, Nana Matsui, Emily Reast, Kaelen Rosander, Carmen Sonnek, Rebecca Wellman, Devin M. Drown

**Affiliations:** a Department of Biology and Wildlife, University of Alaska Fairbanks, Fairbanks, Alaska, USA; b Institute of Arctic Biology, University of Alaska Fairbanks, Fairbanks, Alaska, USA; University of Arizona

## Abstract

Here, we describe the complete genome assemblies of seven *Pseudomonas* sp. isolates collected from a boreal forest soil on the University of Alaska Fairbanks campus. Using the VolTRAX v2 multiplex library preparation for Nanopore sequencing and Illumina reads for polishing, we assembled complete genome sequences for each of the isolates.

## ANNOUNCEMENT

We collected soil from the University of Alaska Fairbanks campus, Alaska (64.859°N, 147.855°W). We sampled this site as part of a multiweek workshop for undergraduates with the goal of isolating, screening, and characterizing subarctic soil bacteria with antibiotic activity against ESKAPE (Enterococcus faecium, Staphylococcus aureus, Klebsiella pneumoniae, Acinetobacter baumannii, Pseudomonas aeruginosa, and *Enterobacter* spp.) pathogen relatives. The ESKAPE pathogens are the leading cause of nosocomial infections worldwide and a major public health threat due to their ability to rapidly develop multidrug resistance to the safest antibiotics used clinically (1). Discovering novel antibiotics that act on the ESKAPE pathogens is therefore critical in tackling the global antibiotic resistance crisis. Although *Pseudomonas* spp. found in soils have been previously established as prolific antibiotic producers (2), the isolates described here originated from subarctic soils that are not well characterized and therefore may be a segue into the discovery of novel antibiotic biosynthesis with activity against ESKAPE pathogens.

We homogenized two 10-cm soil cores and added 1 g of soil to 9 ml tryptic soy broth (TSB). To ensure discrete colony formation, we plated 3 dilutions (1:10, 1:100, 1:1,000) onto tryptic soy agar and used the streak plate method 3 times to purify our randomly selected isolates. Here, we describe 7 isolates that we determined to be *Pseudomonas* spp. To obtain DNA for sequencing for each isolate, we inoculated liquid cultures of TSB, incubated at 22°C overnight, and used 1.8 ml as input for the DNeasy UltraClean microbial kit (Qiagen).

We used a total of 583.5 ng of DNA (range, 19.7 to 224 ng; Table 1) as input for the VolTRAX v2 (Oxford Nanopore Technologies [ONT]) to prepare a barcoded sequencing library (VSK-VMK002 workflow, cartridge ID VAB59563). We sequenced the prepared library using a MinION device (ONT) on an r9.4.1 flow cell (FLO-MIN106, flow cell ID FAK97975) for 72 h (VMK002 script). We base called the raw data using Guppy v3.4.5 (ONT) specifying the high-accuracy model (-c dna_r9.4.1_450bps_hac.cfg) and default parameters. This run generated a total of 14,168,620,548 bp in 2,963,836 reads with an *N*_50_ read length of 6,282 bp. We demultiplexed isolate samples with the guppy_barcoder function of Guppy with parameters to discard sequences with middle adapters (–detect_mid_strand_barcodes) and trim barcodes (–trim_barcodes). We used Filtlong v0.2.0 (https://github.com/rrwick/filtlong) to filter by length (≥50 bp; –min_length 50) and quality (Q) score (≥10; –min_mean_q 90). We assembled the genome sequence for each isolate using Flye v2.7 (3) with default parameters specifying the estimated genome size (-genomesize = 5m) and Nanopore reads (-nanopore-raw) and subsampling for initial disjointing assembly (–asm-coverage 100). The Microbial Genome Sequencing Center (Pittsburgh, PA) prepared a multiplex Nextera library using the previously extracted DNA for sequencing on an Illumina NextSeq 550 to generate paired-end reads (2 × 150 bp). Each individual strain was separately indexed. We used the unicycler_polish tool of Unicycler v0.4.8 (4) for genome polishing. As input, we included the Illumina reads and the Flye assembly. In this mode, Unicycler runs multiple rounds of polishing with Pilon v1.22 (5). Table 1 contains a summary of each of our complete genome sequences.

**TABLE 1:** Summary of sequencing data statistics

Isolate	Input DNA (ng)	Barcode	ONT yield (bp)	No. of ONT reads	Avg length (bp)	Coverage (×)	Illumina yield (bp)	No. of Illumina reads	Genome size (bp)	GC%	No. of contigs	*N*_50_ (bp)	No. of tRNAs	No. of rRNAs	No. of CDSs^*a*^	GenBank accession no.
ADAK2	19.7	BC08	337,234,174	84,274	4,001.64	47	445,101,405	1,739,815	7,105,260	59.6	1	7,105,260	72	19	6,548	CP052862
ADAK7	26.8	BC04	877,616,940	239,767	3,660.29	124	474,255,253	1,827,970	7,105,297	59.6	1	7,105,297	72	19	6,546	CP052861
ADAK13	75.6	BC03	1,820,660,366	357,747	5,089.24	254	389,799,732	1,488,149	7,300,098	60.95	1	7,300,098	67	16	6,704	CP052860
ADAK18	55.8	BC06	575,305,360	144,686	3,976.23	88	379,821,480	1,445,569	6,471,780	59.21	1	6,471,780	66	16	5,872	CP052859
ADAK20	69.6	BC07	951,937,436	177,124	5,374.41	153	361,587,647	1,363,699	6,003,946	60.7	1	6,003,946	68	19	5,389	CP052858
ADAK21	224.0	BC01	1,307,688,884	180,133	7,259.57	227	368,641,802	1,382,086	6,003,863	60.69	1	6,003,863	68	19	5,389	CP052857
ADAK22	112.0	BC02	1,332,557,628	272,239	4,894.81	201	308,344,076	1,155,182	6,509,129	60.71	1	6,509,129	66	16	5,989	CP052856

a CDSs, coding DNA sequences.

We used PATRIC v3.6.3 (6) for initial genome annotation and to extract the 16S rRNA gene sequences for each isolate. To assign taxonomy, we used BLASTn (7) against the NCBI 16S rRNA database. For each isolate, the top five hits (ranked by bit score) included members of the *Pseudomonas* genus. Therefore, we assigned each isolate as a member of that genus. The whole-genome sequences deposited in GenBank were annotated with PGAP (8) as part of the submission pipeline.

### Data availability.

This genome project is indexed at GenBank under BioProject accession number PRJNA627971. These whole-genome sequences have been deposited in GenBank under accession numbers CP052856 through CP052862. Direct links are listed in Table 1. The raw sequencing data for this project can be found in the NCBI SRA under accession number PRJNA627971.
